# A 2D/2D heterojunction of ultrathin Pd nanosheet/MXene towards highly efficient methanol oxidation reaction: the significance of 2D material nanoarchitectonics†

**DOI:** 10.1039/d3sc03735e

**Published:** 2023-09-04

**Authors:** Huajie Huang, Di Xiao, Zihan Zhu, Chi Zhang, Lu Yang, Haiyan He, Jungmok You, Quanguo Jiang, Xingtao Xu, Yusuke Yamauchi

**Affiliations:** a College of Mechanics and Materials, Hohai University Nanjing 210098 China jiangqg@hhu.edu.cn; b Department of Plant & Environmental New Resources, College of Life Sciences, Kyung Hee University 1732 Deogyeong-daero, Giheung-gu Yongin-si Gyeonggi-do 17104 South Korea; c Marine Science and Technology College, Zhejiang Ocean University Zhoushan 316022 China xingtao.xu@zjou.edu.cn; d Australian Institute for Bioengineering and Nanotechnology (AIBN), The University of Queensland Brisbane QLD 4072 Australia y.yamauchi@uq.edu.au; e Department of Materials Process Engineering, Graduate School of Engineering, Nagoya University, Nagoya University Nagoya 464-8601 Japan

## Abstract

Two-dimensional (2D) Pd nanosheet-based catalysts have recently garnered widespread attention due to their high atom utilization efficiency. However, their catalytic ability and structural stability still require significant enhancement before they can be widely applied. In this study, we presented the rational design and controllable fabrication of a novel 2D/2D heterojunction, which consists of ultrathin Pd nanosheets (NSs) grown on the Ti_3_C_2_T_*x*_ MXene surface (Pd NSs/MXene). This heterostructure was achieved through a robust and convenient stereo-assembly strategy. The newly developed Pd NSs/MXene heterojunction not only provides numerous exposed active Pd atoms with an optimized electronic structure but also enables an intimate Pd/MXene interfacial interaction, ensuring a stable hybrid configuration. Consequently, the resulting Pd NSs/MXene heterojunction exhibits exceptional methanol oxidation properties. It possesses a large electrochemically active surface area, high mass and specific activities, and a long operating life, which are significantly superior to those of traditional Pd nanoparticle/carbon and Pd nanosheet/carbon catalysts. Theoretical simulations further reveal strong electronic interactions between the Pd nanosheet and MXene, which dramatically enhance the adsorption energy of the Pd component and simultaneously lower its d-band center. As a result, the Pd NSs/MXene heterojunction is less susceptible to CO poisoning. This work introduces a new 2D/2D heterojunction based on MXene and noble metallic materials and holds significance for the development of other novel heterojunctions, particularly within the realm of 2D material nanoarchitectonics.

## Abstract 2

The stereo-assembly of ultrathin Pd nanosheets *in situ* grown on the MXene surface was achieved *via* a convenient wet-chemical approach, which possess superior electrocatalytic performance for the methanol oxidation reaction.

## Graphical abstract

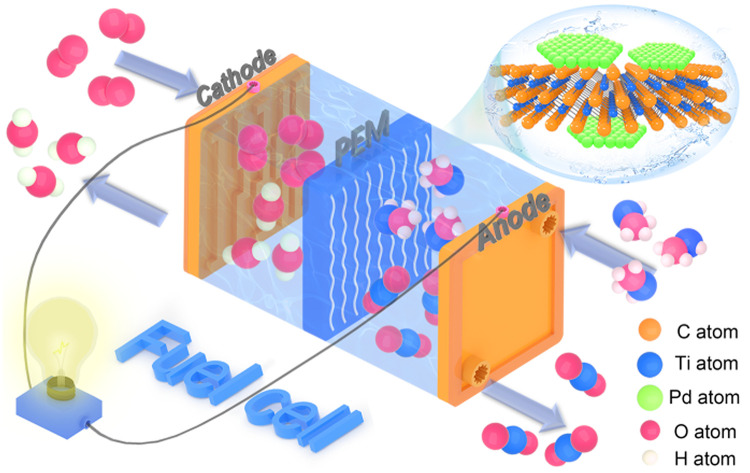

## 1. Introduction

The rapidly growing energy demand worldwide has exerted tremendous pressure on energy supply and the ecological environment. This pressure has spurred the development of various advanced energy technologies, including fuel cells, photocatalysis, and capacitive deionization.^1–5^ Among these technologies, the direct methanol fuel cell (DMFC) stands out as a green and highly efficient power source due to its unique advantages, such as a wide operating temperature range, low pollutant emissions, simple device construction, and convenient storage of liquid methanol fuel.^6–8^ However, the inherently sluggish kinetics of the methanol oxidation severely restricts the overall energy and power densities of DMFCs.^9–11^ This limitation emphasizes the crucial role of selecting suitable electrode materials in enhancing the reaction rate.

Currently, platinum (Pt) and Pt-based materials are commonly used as anode catalysts for DMFCs. However, the poor tolerance of Pt towards CO poisoning presents significant challenges for its commercial applications.^12–14^ Therefore, extensive efforts have been dedicated to exploring and utilizing efficient non-Pt catalysts with enhanced anti-poisoning properties.^15–18^ In this regard, metallic palladium (Pd) has garnered considerable and sustained attention due to its superior resistance to CO species compared to Pt in alkaline electrolytes, which can provide more sustainable electrocatalytic activity.^19–23^ Furthermore, it has been demonstrated that two-dimensional (2D) Pd nanocrystals with a thin-sheet structure exhibit improved electrocatalytic performance compared to conventional zero-dimensional (0D) Pd nanoparticles (NPs). This improvement is attributed to their high surface-to-volume ratio and high-density of unsaturated atoms.^24–26^ However, the presence of a significant proportion of low-coordinated Pd atoms with high surface energy makes Pd nanosheets susceptible to various structural changes, such as agglomeration, dissolution, and Ostwald ripening.^27–29^ These structural evolutions can lead to a decline in catalytic stability.

To address the aforementioned issue, one potential solution is to grow Pd nanocrystals on a robust high-quality matrix with a large specific surface area and excellent chemical stability.^30,31^ It is well known that commercial Pd catalysts often employ carbonaceous supports to stabilize the Pd structure, thereby reducing the amount of Pd used and improving its utilization efficiency.^32,33^ However, most conventional carbon supports (such as carbon black, carbon nanotubes, graphene, *etc.*) are electrochemically inert due to their high graphitization degree, making it challenging for them to directly participate in electrocatalytic processes.^34,35^ Moreover, the surfaces of these carbon supports lack efficient anchoring sites for nucleation and immobilization of Pd nanocrystals, limiting the full realization of synergistic coupling effects.^36^ In recent years, 2D MXenes have emerged as a hotspot in the field of electrocatalysis due to their ultrathin nature, tunable chemistries, abundant functional groups, and exceptional metallic conductivity.^37–39^ Our recent experimental and theoretical investigations have demonstrated that 2D Ti_3_C_2_T_*x*_ MXene, as a novel matrix, can ensure the uniform dispersion of noble metal nanocrystals while optimizing their electronic structures to achieve high catalytic efficiency.^40,41^ However, it is important to note that the random distribution of terminal functional groups (*e.g.*, –O, –F, –OH) on Ti_3_C_2_T_*x*_ MXene often leads to the formation of particle-shaped Pd structures.^42,43^ Therefore, the *in situ* oriented growth of 2D Pd nanosheets on the MXene surface still poses a significant challenge in this emerging field.

**Fig. 1:**
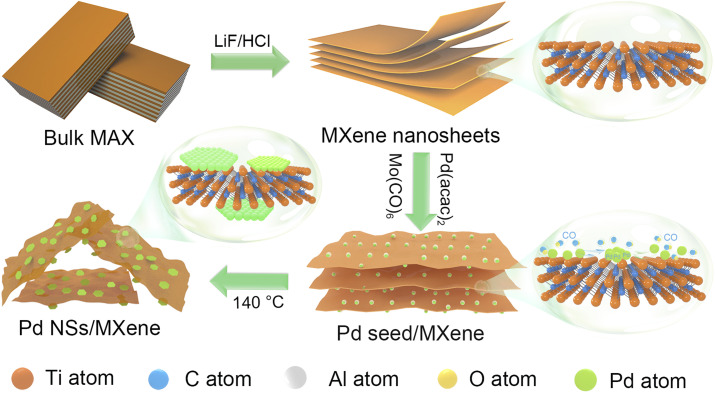
The synthesis process of the 2D/2D Pd NSs/MXene heterojunction: (1) synthesis of 2D MXene nanolayers by a LiF and HCl etching route; (2) growth of Pd seeds on the surface of MXene as well as the generation of CO molecules by the decomposition of Mo(CO)_6_; (3) gradual formation of 2D Pd nanosheets on the MXene nanosheets.

In this study, we present a robust and convenient wet-chemical approach for synthesizing a 2D/2D heterojunction consisting of ultrathin Pd nanosheets grown *in situ* on MXene (Pd NSs/MXene) through a straightforward stereo-assembly process. The synthesis procedure is illustrated in Fig. 1. First, 2D Ti_3_C_2_T_*x*_ MXene nanosheets were produced by etching the commercial Ti_3_AlC_2_ material in the presence of LiF and HCl. The resulting Ti_3_C_2_T_*x*_ material was then suspended in a 1-methyl-2-pyrrolidone (NMP) solution through ultrasonication, forming a uniform black dispersion. Next, suitable amounts of Pd precursor (Pd(acac)_2_), Mo(CO)_6_, and acetic acid were added to the Ti_3_C_2_T_*x*_ dispersion and thoroughly mixed using ultrasonication. The resulting mixture was subsequently heated at 140 °C for 1 hour in an oil bath. During this process, the decomposition of Mo(CO)_6_ and the moderate reducing environment facilitated the lateral-directional growth of ultrathin Pd nanosheets on the Ti_3_C_2_T_*x*_ nanoflakes, leading to the formation of the 2D/2D Pd NSs/MXene heterojunction. The resulting Pd NSs/MXene heterojunction exhibited unique architectural features, including a large accessible surface area, intimately contacted interface, numerous unsaturated Pd atoms, modified electronic structures, and high electrical conductivity. These features contributed to significantly enhanced electrocatalytic properties for methanol oxidation compared to the reference Pd nanoparticle/carbon and Pd nanosheet/carbon catalysts. Consequently, the Pd NSs/MXene heterojunction holds great promise as an advanced anode material for high-performance DMFC devices.

## 2. Experimental

### 2.1. Synthesis of the 2D/2D Pd NSs/MXene heterojunction

First, 2D high-quality Ti_3_C_2_T_*x*_ MXene nanosheets were synthesized from the commercial Ti_3_AlC_2_ material (Fig. S1†) using a wet etching approach with the assistance of LiF and HCl.^40^ Subsequently, 20 mg of the prepared Ti_3_C_2_T_*x*_ nanosheets were placed in a vial containing 10 mL of NMP and subjected to ultrasonic treatment for 1 hour to obtain a uniform black colloidal solution (Fig. S2 and S3†). Next, 14 mg of Pd(acac)_2_, 5 mg of Mo(CO)_6_, and 2 mL of acetic acid were added sequentially to the Ti_3_C_2_T_*x*_ suspension, followed by another 30 minute ultrasonic treatment. The mixture was then heated at 140 °C for 1 hour in an oil bath. The resulting product, named Pd NSs/MXene, was washed with deionized water and ethanol multiple times and finally dehydrated by lyophilization to prevent nanosheet restacking. Additionally, the same synthesis method was used to prepare Pd nanosheet catalysts supported by reduced graphene oxide (Pd NSs/RGO), carbon nanotubes (Pd NSs/CNT), and carbon black (Pd NSs/C), with the MXene support replaced by the corresponding carbon materials. Meanwhile, conventional Pd NPs supported by various matrices were also fabricated and named Pd NPs/MXene, Pd NPs/RGO, Pd NPs/CNT, and Pd NPs/C. In detail, 20 mg of carbon matrix was fully dispersed in 40 mL of a 50% ethylene glycol solution through sonication. Then, 14 mg of Pd(acac)_2_ was added under magnetic stirring. The resulting sample was transferred to a Teflon autoclave and maintained at 140 °C for 12 hours, yielding the desired reference samples. The actual Pd contents in Pd NSs/MXene, Pd NPs/MXene, Pd NPs/RGO, Pd NPs/CNT, and Pd NPs/C, as determined by inductively coupled plasma mass spectrometer (ICP-MS) tests, were found to be 18.7 wt%, 19.0 wt%, 18.5 wt%, 17.9 wt%, and 18.6 wt%, respectively.

### 2.2. Characterization

The 2D/2D heterostructure and surface morphology of the Pd NSs/MXene catalyst were examined using field emission scanning electron microscopy (FE-SEM, JEOL 6500F), transmission electron microscopy (TEM, JEOL 2100F), and atomic force microscopy (AFM, Nanoscope IIIA). The crystal structure of Pd NSs/MXene was investigated through powder X-ray diffraction (XRD) tests using a Bruker D8 Advance instrument. The elemental composition information of Pd NSs/MXene was obtained through X-ray photoelectron spectroscopy (XPS) measurements conducted with a Thermo ESCALAB 250 spectrometer. Ultraviolet-visible (UV-vis) absorption spectra were collected using a TU-1901 spectrophotometer. The Pd contents of the various catalysts were determined using a PerkinElmer ELAN9000 ICP-MS.

### 2.3. Electrochemical measurements

The methanol oxidation tests for the Pd NSs/MXene and reference catalysts were conducted using a CHI 760E electrochemical workstation with a standard three-electrode operating system. The system consisted of a Pt wire, a saturated calomel electrode (SCE), and a 3 mm glass carbon disk, serving as the counter, reference, and working electrodes, respectively. The detailed manufacturing processes for the working electrode and the electrochemical techniques have been previously described in our earlier work.^38^ The electrochemical measurements were performed in an alkaline electrolyte solution purged with nitrogen, which consisted of 0.5 M NaOH and 1 M CH_3_OH.

### 2.4. Density functional theory (DFT) calculations

A 4 × 3 Ti_3_C_2_(OH)_*x*_ supercell was employed, and the *k*-point mesh was set to 6 × 8 × 1 for structure relaxations. The convergence tolerance for energy was set to 10^−5^ Hartree, while the maximum allowed force and displacement were set to 0.002 Hartree per Å and 0.005 Å, respectively. The generalized gradient approximation (GGA) with the Perdew–Burke–Ernzerhof (PBE) functional was utilized for the exchange-correlation functional.

## 3. Results and discussion

**Fig. 2:**
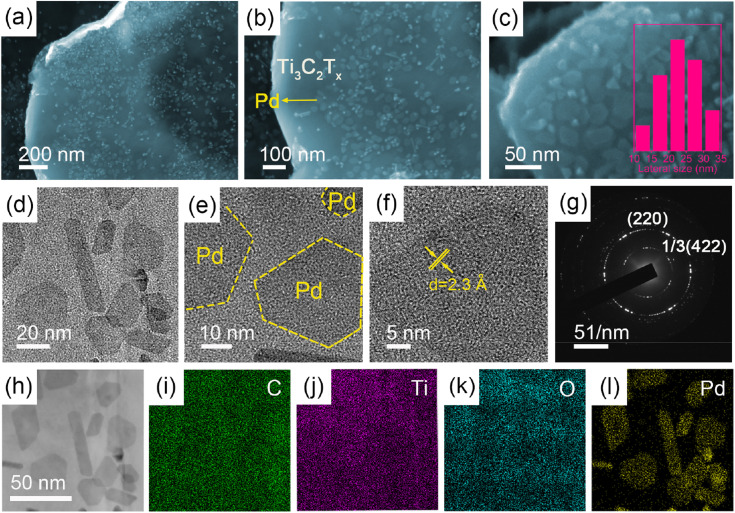
FE-SEM and TEM analysis of the Pd NSs/MXene heterojunction. Typical (a–c) FE-SEM, and (d and e) TEM images show that numerous Pd nanosheets are evenly distributed on the Ti_3_C_2_T_*x*_ nanosheets. The inset in (c) is the corresponding Pd lateral size distribution. (f) HR-TEM image and (g) SAED pattern reveal the detailed crystal texture for Pd NSs/MXene. (h) STEM image and elemental analysis confirm that the (i) C, (j) Ti, (k) O, and (l) Pd elements are uniformly dispersed in the heterojunction.

The morphology and nanostructure of the 2D/2D Pd NSs/MXene heterojunction were initially analyzed using FE-SEM and TEM techniques. Fig. 2a shows the exfoliated Ti_3_C_2_T_*x*_ MXene nanosheets, which exhibit a typical 2D thin-sheet structure in contrast to the original bulk structure of the Ti_3_AlC_2_ material (Fig. S1†). Furthermore, a significant number of tightly attached ultrathin Pd NSs are observed on the surface of Ti_3_C_2_T_*x*_ through face-to-face contact, effectively preventing Pd from re-aggregation or restacking (Fig. 2b). At higher magnification, it is evident that the Pd NSs have a hexagonal shape and lateral sizes ranging from 10 to 35 nm, providing abundant unsaturated Pd atoms for additional catalytic activity (Fig. 2c–e). The UV-vis absorption spectrum of Pd NSs/MXene, shown in Fig. S4,† exhibits a prominent peak at around 860 nm, corresponding to the surface plasmon resonance (SPR) feature of 2D Pd nanosheets. AFM analysis reveals that the thickness of the Pd NSs grown on MXene is approximately 2 nm (Fig. S5†), indicating that they are less than 10 atomic layers thick. However, in the case of the reference samples, namely Pd NPs/C, Pd NPs/CNT, Pd NPs/RGO, and Pd NPs/MXene, the Pd nanocrystals are randomly distributed and interact with the matrices in a particle-to-face mode, resulting in the formation of large Pd NPs with limited catalytically active sites (Fig. S6†). High-resolution TEM (HR-TEM) and selected-area electron diffraction (SAED) analyses reveal the typical lattice fringes with an interplanar spacing of 2.3 Å on a single Pd nanosheet (Fig. 2f and g), in good agreement with the 1/3(422) fringes of the face-centered cubic (FCC) Pd structure. This implies that the dominant exposed crystal faces of the Pd NSs are (111) facets, primarily due to the adsorption of CO molecules on the basal (111) faces during the synthetic process.^44^ Moreover, scanning TEM (STEM) imaging and elemental mapping analysis demonstrate that the Pd NSs/MXene heterojunction consists of C, Ti, O, and Pd elements, which are homogeneously dispersed across the nearly transparent MXene nanosheets (Fig. 2h–l).

**Fig. 3:**
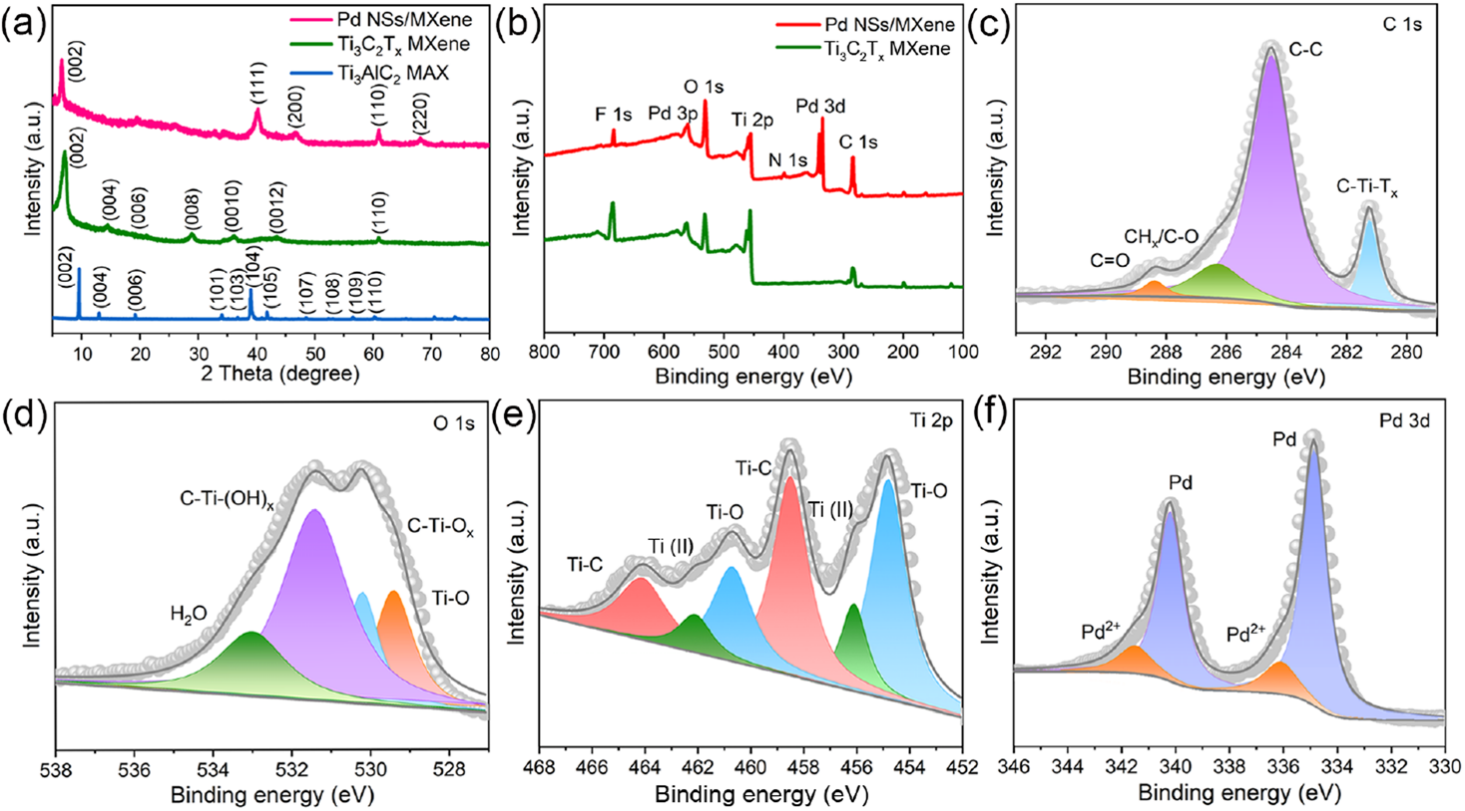
Structural characterization of the Pd NSs/MXene heterojunction. (a) XRD patterns of the Pd NSs/MXene, Ti_3_C_2_T_*x*_ MXene, and Ti_3_AlC_2_ samples. (b) XPS survey spectra of Pd NSs/MXene and Ti_3_C_2_T_*x*_ MXene. The typical (c) C 1s, (d) O 1s, (e) Ti 2p, and (f) Pd 3d core-level XPS spectra of Pd NSs/MXene.

To determine the specific crystalline phases of the Pd NSs/MXene heterojunction in more detail, powder XRD measurements were performed. As depicted in Fig. 3a, the XRD pattern of the delaminated Ti_3_C_2_T_*x*_ nanosheets exhibits a prominent diffraction peak at around 2*θ* = ∼7.0°, corresponding to the (002) plane. In contrast, the (104) peak observed in bulk Ti_3_AlC_2_ at 2*θ* = 39.1° is no longer present, confirming the successful etching and exfoliation of the Ti_3_AlC_2_ material.^45,46^ Upon the growth of ultrathin Pd nanosheets on the Ti_3_C_2_T_*x*_ nanosheets, a series of characteristic Pd peaks are observed at 2*θ* = 39.8°, 46.6°, and 67.8°. These peaks can be attributed to the (111), (200), and (220) crystal planes of FCC Pd crystals, respectively.

XPS analysis was performed to investigate the elemental composition and chemical states of the Pd NSs/MXene heterojunction. The XPS survey spectrum of the Pd NSs/MXene heterojunction, as shown in Fig. 3b, exhibits several prominent elemental signals, including Pd 3d, Ti 2p, C 1s, O 1s, and F 1s, which align well with the energy dispersive X-ray (EDX) results (Fig. S7†). Fig. 3c presents the deconvolution of the high-resolution C 1s spectrum, revealing four distinct peaks at binding energies of 281.4, 284.5, 286.3, and 288.5 eV, corresponding to the C–Ti–T_*x*_, C–C, CH_*x*_/C–O, and CO groups, respectively. Additionally, the O 1s signal is deconvoluted into four peaks at 529.4, 530.2, 531.4, and 533.0 eV, associated with Ti–O, C–Ti–O_*x*_, C–Ti–(OH)_*x*_, and H_2_O species, respectively (Fig. 3d). Moreover, the complex Ti 2p spectrum can be deconvoluted into multiple peaks at 454.8 (460.7), 456.1 (462.1), and 458.5 (464.1) eV, corresponding to the Ti–O, Ti^2+^, and Ti–C configurations, respectively (Fig. 3e).^47,48^ The Pd 3d spectral fitting yields two pairs of energy peaks: the two intense peaks centered at 334.9 and 340.2 eV correspond to metallic Pd with a zero valence state, while the other two weaker peaks at 336.1 and 341.5 eV are associated with divalent Pd oxide (Fig. 3f). Notably, compared to the bare Pd NSs sample, the binding energies of the metallic Pd peaks in the Pd NSs/MXene heterojunction exhibit a negative shift of approximately ∼0.4 eV (Fig. S8†). This shift suggests the presence of a well-contacted 2D/2D interface, facilitating direct electron transfer between the Pd NSs and MXene matrix.

**Fig. 4:**
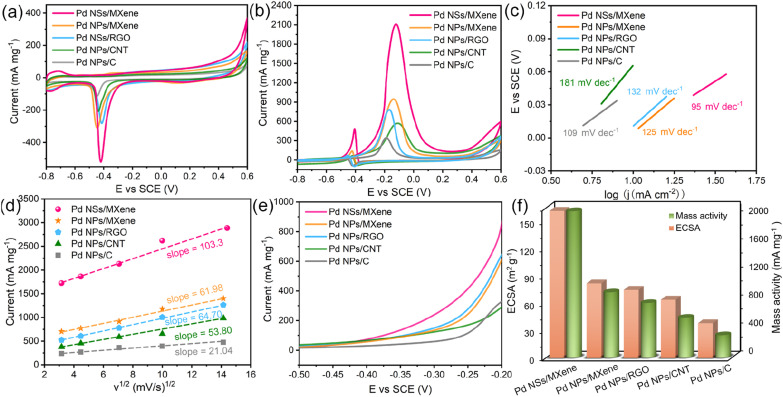
Electrocatalytic activity of the Pd NSs/MXene heterojunction towards the methanol oxidation reaction. CV curves of the Pd NSs/MXene, Pd NPs/MXene, Pd NPs/RGO, Pd NPs/CNT, and Pd NPs/C electrodes in (a) 0.5 M NaOH solution and (b) 0.5 M NaOH with 1 M CH_3_OH solution at 50 mV s^−1^. (c) The corresponding Tafel plots and (d) the relationship of the peak current to the square root of the scan rate for various electrodes. (e) LSV curves of the Pd NSs/MXene, Pd NPs/MXene, Pd NPs/RGO, Pd NPs/CNT, and Pd NPs/C electrodes in 0.5 M NaOH with 1 M CH_3_OH solution at 50 mV s^−1^. (f) Specific ECSA values and mass activities for various electrodes.

The sophisticated 2D/2D nanoarchitecture of the Pd NSs/MXene heterojunction has inspired us to explore its potential as an anode catalyst for DMFCs and evaluate its electrocatalytic performance for the methanol oxidation reaction (Fig. 4a–f). For comparison, we also investigated the performance of Pd NPs/MXene, Pd NPs/RGO, Pd NPs/CNT, and Pd NPs/C catalysts. Cyclic voltammetry (CV) curves of the various electrodes were recorded in a N_2_-saturated 0.5 M NaOH electrolyte, as shown in Fig. 4a. A distinct backward current peak, associated with the reduction of Pd oxides, is observed around −0.45 V. This peak is commonly used to estimate the electrochemically active surface area (ECSA) of the Pd catalysts. Based on the integration of the current peak area, the ECSA value of the Pd NSs/MXene electrode is calculated to be 162.0 m^2^ g^−1^, which is significantly larger than that of the Pd NPs/MXene (82.2 m^2^ g^−1^), Pd NPs/RGO (75.0 m^2^ g^−1^), Pd NPs/CNT (64.2 m^2^ g^−1^), and Pd NPs/C (41.2 m^2^ g^−1^) electrodes (Table S1† and Fig. 4f). This result suggests the presence of a higher abundance of accessible Pd sites in the Pd NSs/MXene heterostructure. Furthermore, the ECSA value obtained for the Pd NSs/MXene catalyst is competitive with those of recent state-of-the-art Pd-based catalysts, Pd/functionalized CNT,^49,50^ Pd/heteroatom-doped graphene,^51,52^ Pd/metal oxide-modified graphene,^53,54^ Pd/porous carbon,^31,33^ Pd-based nanodendrites,^55,56^ Pd alloy nanowires,^57,58^ and others (Table S2†). These findings highlight the potential of the newly-designed Pd NSs/MXene heterojunction for future applications in the field of fuel cells.

The methanol oxidation tests were conducted in a mixed electrolyte containing 0.5 M NaOH and 1 M CH_3_OH. Fig. 4b illustrates the recorded CV curves, which exhibit a clear anodic peak at around −0.15 V and a weak cathodic peak at −0.40 V. These peaks correspond to the oxidation of fresh methanol molecules and CO-like by-products, respectively. The anodic peak current densities can be used to evaluate the methanol oxidation mass activity of the different electrodes. Notably, the Pd NSs/MXene electrode demonstrates an exceptionally high methanol oxidation mass activity of up to 2091.4 mA mg^−1^, followed by the Pd NPs/MXene (934.1 mA mg^−1^), Pd NPs/RGO (780.9 mA mg^−1^), Pd NPs/CNT (569.9 mA mg^−1^), and Pd NPs/C (345.5 mA mg^−1^) electrodes, aligning with the earlier observed ECSA trend. Furthermore, both the ECSA value and mass activity of the Pd NSs/MXene electrode surpass those of Pd NSs/C, Pd NSs/CNT, and Pd NSs/RGO (Fig. S9 and Table S1†), indicating the superior catalytic capacity of the MXene support. The Tafel slope of the Pd NSs/MXene electrode is determined to be 95 mV dec^−1^, considerably smaller than that of the other reference electrodes (109–181 mV dec^−1^), suggesting a higher methanol oxidation efficiency with the use of the Pd NSs/MXene catalyst (Fig. 4c). Additionally, CV curves of the aforementioned Pd-based electrodes were collected at varying scan rates ranging from 10 to 200 mV s^−1^. As depicted in Fig. 4d and S10,† the methanol oxidation peak currents for all electrodes exhibit a linear relationship with the square root of the scan rates, indicating a diffusion-controlled catalytic process.^59^ Notably, the steepest slope is observed for the Pd NSs/MXene electrode, implying the highest electron transfer coefficient in the kinetic reaction. Moreover, the results of linear-sweep voltammetry (LSV) measurements demonstrate that the Pd NSs/MXene electrode achieves the same oxidation current as the comparison electrodes at a significantly lower electrode potential (Fig. 4e). This confirms that the presence of the Pd NSs/MXene catalyst effectively reduces the activation energy for the methanol oxidation process. To further analyze the mass activities of the different electrodes, they were normalized by their respective ECSA values. As shown in Fig. S11 and Table S1†, the ECSA-normalized specific activity of the Pd NSs/MXene electrode (1.30 mA cm^−2^) is the highest among those of the other electrodes, indicating its superior intrinsic catalytic ability for methanol electrooxidation.

**Fig. 5:**
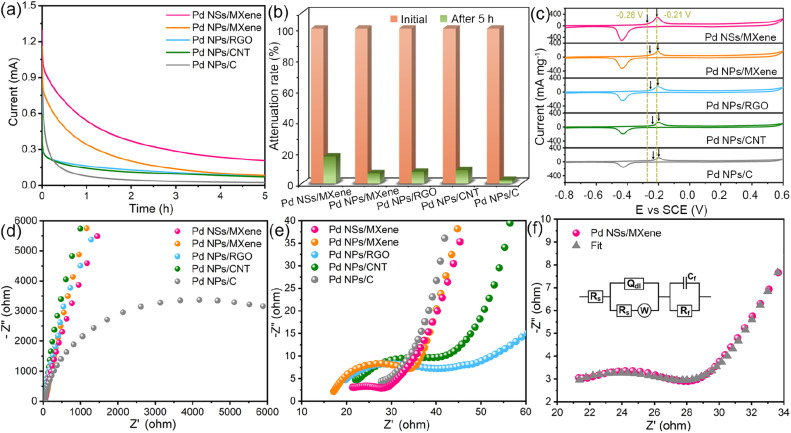
(a) Chronoamperometric curves of the Pd NSs/MXene, Pd NPs/MXene, Pd NPs/RGO, Pd NPs/CNT, and Pd NPs/C electrodes in 0.5 M NaOH and 1 M CH_3_OH solution. (b) Mass activities of various electrodes before and after chronoamperometry measurements. (c) CO stripping voltammograms of the Pd NSs/MXene, Pd NPs/MXene, Pd NPs/RGO, Pd NPs/CNT, and Pd NPs/C electrodes in 0.5 M NaOH solution at 50 mV s^−1^. (d and e) AC impedance spectra of the Pd NSs/MXene, Pd NPs/MXene, Pd NPs/RGO, Pd NPs/CNT, and Pd NPs/C electrodes. (f) AC impedance spectrum and the corresponding fitting curve of the Pd NSs/MXene electrode. The inset in (f) is the equivalent circuit used to fit the impedance spectra.

A long service life is a crucial requirement for the practical application of a fuel cell electrocatalyst. In this regard, the long-term stability of the Pd NSs/MXene catalyst was investigated using chronoamperometric measurements over a period of 5 hours. Under a constant electrode potential of −0.2 V, the methanol oxidation currents on all examined electrodes initially decline rapidly and then gradually reach a pseudo-equilibrium state (Fig. 5a). Remarkably, the Pd NSs/MXene electrode not only maintains a significantly higher oxidation current but also exhibits a slower decay rate compared to the other electrodes throughout the entire testing process, thereby demonstrating a more reliable long-term durability (Fig. 5b).

Further FE-SEM, TEM, and elemental mapping analysis confirm that the overall integrated 2D/2D architectural features of the Pd NSs/MXene catalyst are well preserved after the long-term durability test (Fig. S12 and S13†). This preservation can be attributed to the strong face-to-face interaction between Pd NSs and MXene, which effectively prevents the heterojunction from undergoing various structural evolutions. Moreover, there are no apparent changes in the composition and chemical valences of the Pd NSs/MXene catalyst observed during the stability test (Fig. S14†), indicating its stable chemical properties.

The close Pd/MXene interfacial interaction is also expected to significantly enhance the catalyst's inherent anti-poisoning capacity for CO-like intermediates. Fig. 5c displays the CO stripping voltammograms for the various electrodes in a 0.5 M NaOH electrolyte. Among them, the Pd NSs/MXene electrode exhibits the lowest onset potential (−0.28 V) and peak potential (−0.21 V) for CO oxidation, suggesting its superior anti-toxic ability arising from the unique 2D/2D heterostructure.

Additionally, the fast charge-transfer rate of the catalytic system may also contribute to the enhanced methanol oxidation efficiency. To investigate this aspect, the alternating-current (AC) impedance technique was employed to compare the electron conductivities of different electrode catalysts. Fig. 5d–e present the collected AC impedance spectra, which exhibit well-defined semicircles in the high-frequency range. The diameters of these semicircles are commonly used to estimate the charge transfer resistances (*R*_ct_) of Pd-based electrocatalysts. Due to its unique thin-sheet Pd structure with face-to-face contact to the highly conductive MXene support, the Pd NSs/MXene catalyst exhibits an extremely low *R*_ct_ value of only 7.9 Ω, significantly outperforming the *R*_ct_ values of Pd NPs/MXene (17.9 Ω), Pd NPs/RGO (21.4 Ω), Pd NPs/CNT (22.1 Ω), and Pd NPs/C (6875.1 Ω) catalysts (Fig. 5f and Table S3†). This low *R*_ct_ value of the Pd NSs/MXene catalyst enables accelerated methanol electrooxidation kinetics on its electrode surface, contributing to its enhanced performance.

**Fig. 6:**
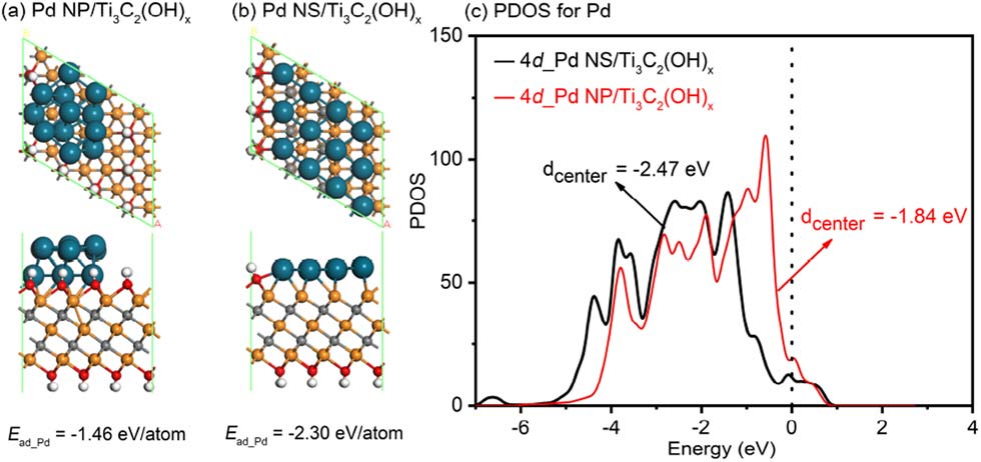
The relaxed atomic structures of the (a) Pd NP/Ti_3_C_2_(OH)_*x*_ and (b) Pd NS/Ti_3_C_2_(OH)_*x*_ models, where the gray, red, white, orange, and blue balls represent C, O, H, Ti, and Pd atoms, respectively. (c) The d-PDOS plots of the different Pd nanostructures in the Pd NP/Ti_3_C_2_(OH)_*x*_ and Pd NS/Ti_3_C_2_(OH)_*x*_ models.

To gain a deeper understanding of the exceptional electrocatalytic performance of the 2D/2D Pd nanosheet/MXene system, spin-polarized DFT calculations were conducted to investigate the synergistic effects between Pd nanostructures and substrates. A typical Pd nanoparticle and a Pd nanosheet, each consisting of twelve atoms, were studied on the Ti_3_C_2_(OH)_*x*_ model, labeled as Pd NP/Ti_3_C_2_(OH)_*x*_ and Pd NS/Ti_3_C_2_(OH)_*x*_, respectively. The aim was to uncover the detailed atomic-level interfacial interactions. As illustrated in Fig. 6a and b, the adsorption energy for Pd NPs on the Ti_3_C_2_(OH)_*x*_ support is −1.46 eV per atom, whereas for Pd NSs on the Ti_3_C_2_(OH)_*x*_ support, it is −2.30 eV per atom. This confirms that the interaction between Pd NSs and Ti_3_C_2_(OH)_*x*_ is much stronger than that between Pd NPs and Ti_3_C_2_(OH)_*x*_. Furthermore, the d-band center is known to be a suitable indicator for the adsorption ability of noble metals towards small molecules, where a right shift of the d-band center typically signifies a stronger interaction between the metal and the molecules.^60–62^ In this study, the stronger interaction between Pd NSs and Ti_3_C_2_(OH)_*x*_ is expected to result in a left shift of the d-band center for Pd NSs relative to Pd NP, thereby reducing the overlap between the d-band of Pd NSs and the sp-band of the CO molecule. To confirm this hypothesis, the partial density of states (PDOSs) for the Pd NP/Ti_3_C_2_(OH)_*x*_ and Pd NS/Ti_3_C_2_(OH)_*x*_ models was analyzed, as shown in Fig. 6c. The calculated d-band centers were determined to be −1.84 and −2.47 eV for Pd NPs and Pd NSs, respectively. Strikingly, the d-band of Pd NSs on the Ti_3_C_2_(OH)_*x*_ matrix exhibited a significant left shift of approximately 0.64 eV compared to that of Pd NPs on the Ti_3_C_2_(OH)_*x*_ matrix. This provides convincing evidence that the adsorption of CO molecules is more challenging on the 2D/2D Pd NS/Ti_3_C_2_(OH)_*x*_ model.

**Fig. 7:**
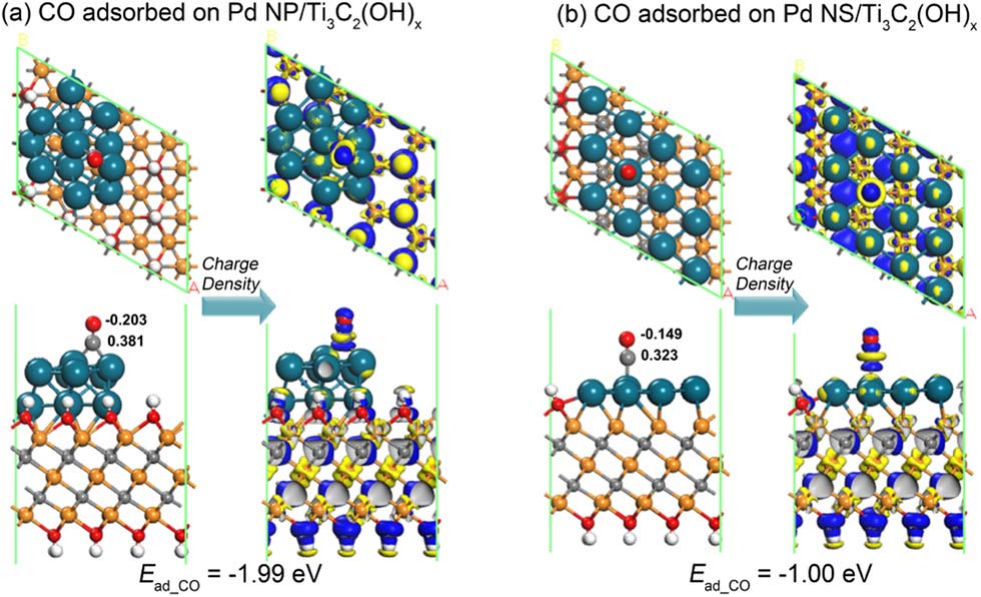
The relaxed atomic structures for the adsorption of a CO molecule on the (a) Pd NP/Ti_3_C_2_(OH)_*x*_ and (b) Pd NS/Ti_3_C_2_(OH)_*x*_ models as well as the corresponding deformation charge density near the CO molecule. The blue and yellow colors indicate the increase and depletion of electrons, respectively.

The significant difference in adsorption abilities between the Pd NP/Ti_3_C_2_(OH)_*x*_ and Pd NS/Ti_3_C_2_(OH)_*x*_ structures is directly evident from their distinct CO adsorption energies. As depicted in Fig. 7, the CO adsorption energy of the Pd NS/Ti_3_C_2_(OH)_*x*_ model (−1.00 eV) is much lower than that of the Pd NP/Ti_3_C_2_(OH)_*x*_ model (−1.99 eV). The corresponding energy difference of 0.99 eV is slightly larger than the previously mentioned left shift of the d-band center by 0.64 eV, suggesting that the d-band center serves as a reliable indicator to describe the affinity of Pd nanostructures for CO molecules. The Mulliken charge distributions near the CO byproduct are also shown in Fig. 7, where the CO molecule loses electrons of 0.178 e and 0.174 e on Pd NP/Ti_3_C_2_(OH)_*x*_ and Pd NS/Ti_3_C_2_(OH)_*x*_, respectively. This further indicates the relatively weaker adsorption of the CO molecule on the latter configuration. Moreover, based on the deformation charge density near the CO molecule, the electron depletion of the CO molecule on the Pd NS/Ti_3_C_2_(OH)_*x*_ model is significantly reduced compared to that on the Pd NP/Ti_3_C_2_(OH)_*x*_ model. Therefore, we can confidently conclude that the stereo-assembly of ultrathin Pd nanosheets on the MXene surface ensures a high resistance to CO byproduct poisoning and simultaneously prevents agglomeration, dissolution, and Ostwald ripening of the Pd component.

## 4. Conclusions

We have developed a robust and straightforward wet-chemical approach for the *in situ* synthesis of a 2D/2D heterojunction comprising ultrathin Pd nanosheets strongly coupled with MXene through a controllable stereo-assembly process. The resulting Pd NSs/MXene heterojunction exhibits several highly desirable architectural features, including a large accessible surface area, well-contacted interface, abundant unsaturated Pd atoms, improved electronic structures, and excellent electrical conductivity. As a result, the 2D/2D Pd NSs/MXene heterojunction demonstrates remarkable methanol oxidation performance, with a large ECSA value of 162.0 m^2^ g^−1^, a high mass activity of 2091.4 mA mg^−1^, and good long-term durability. These results far surpass those of conventional Pd nanoparticle/carbon and Pd nanosheet/carbon catalysts. DFT calculations further reveal that the face-to-face contact induces a strong electronic interaction between the Pd nanosheet and MXene, resulting in an increased Pd adsorption energy and a downward shift of the d-band center, thus enhancing CO poisoning tolerance.

## Data availability

All relevant data supporting this article have been included in the main text and the ESI.† All original data generated during this work are available from the corresponding authors upon request.

## Author contributions

Huajie Huang: conceptualization, methodology, investigation, writing – original draft; Di Xiao: investigation, formal analysis, data curation, writing – original draft; Zihan Zhu: investigation, formal analysis; Chi Zhang: investigation, writing – review and editing; Lu Yang: investigation, visualization; Haiyan He: investigation; Jungmok You: writing – review and editing; Quanguo Jiang: methodology, investigation, formal analysis, data curation, software; Xingtao Xu: formal analysis, investigation, visualization, writing – review and editing; Yusuke Yamauchi: resources, supervision, writing – review and editing.

## Conflicts of interest

There are no conflicts to declare.

## Supplementary Material

SC-014-D3SC03735E-s001
