# Supplementary material for: A 2D/2D heterojunction of ultrathin Pd nanosheet/MXene towards highly efficient methanol oxidation reaction: the significance of 2D material nanoarchitectonics
Source: Chem Sci. 2023 Sep 4;14(36):9854–62. doi: 10.1039/d3sc03735e (PMC10510762; doi:10.1039/d3sc03735e)
Supplement: SC-014-D3SC03735E-s001 [file SC-014-D3SC03735E-s001.pdf]

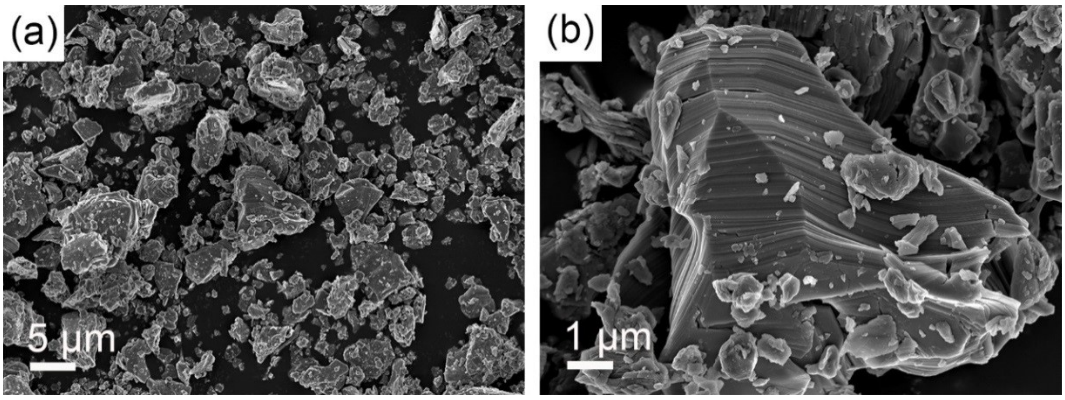

Fig. S1 Representative FE-SEM images of Ti_3AlC_2 powders at different magnifications.

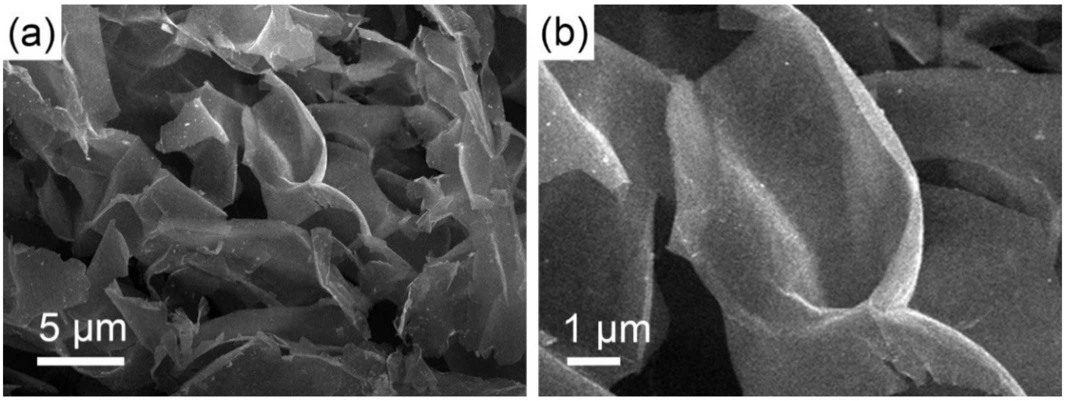

Fig. S2 Representative FE-SEM images of exfoliated $Ti_3C_2T_x$ nanosheets show their 2D layer structure.

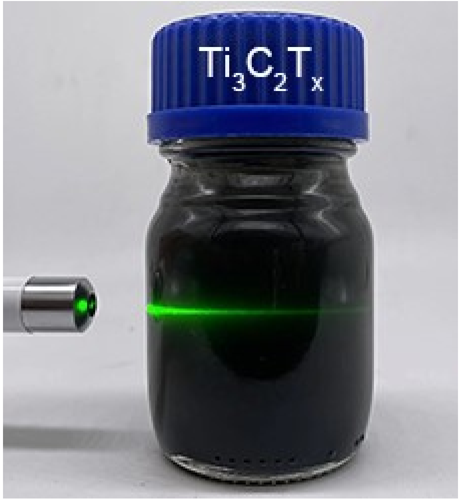

Fig. S3 The Tyndall phenomenon of the as-obtained $Ti_3C_2T_x$ MXene suspension.

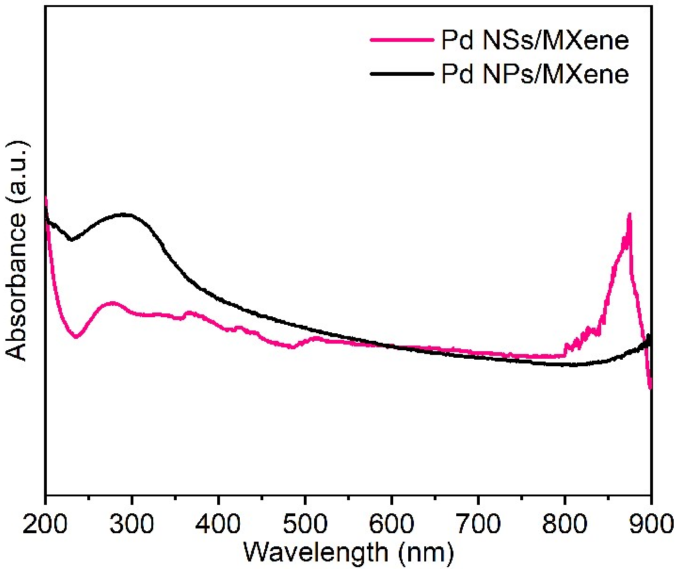

Fig. S4 UV-vis absorption spectra of the Pd NSs/MXene and Pd NPs/MXene samples.

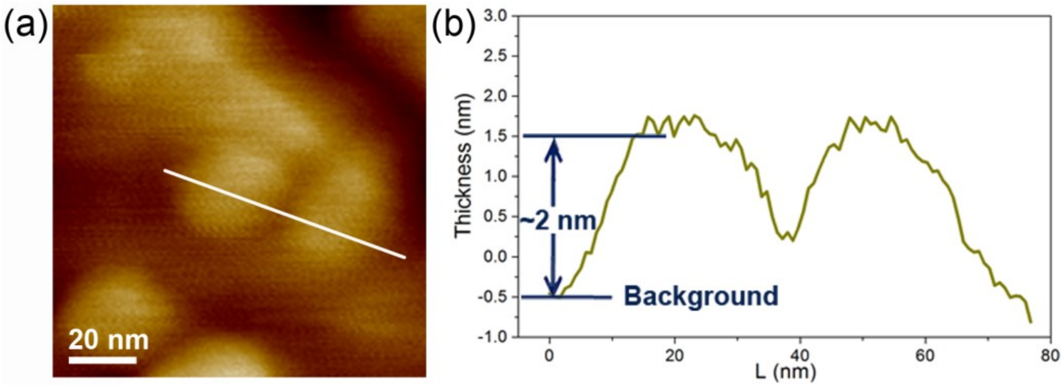

Fig. S5 (a) AFM image of the Pd NSs/MXene heterojunction. (b) The thickness analysis along the white line.

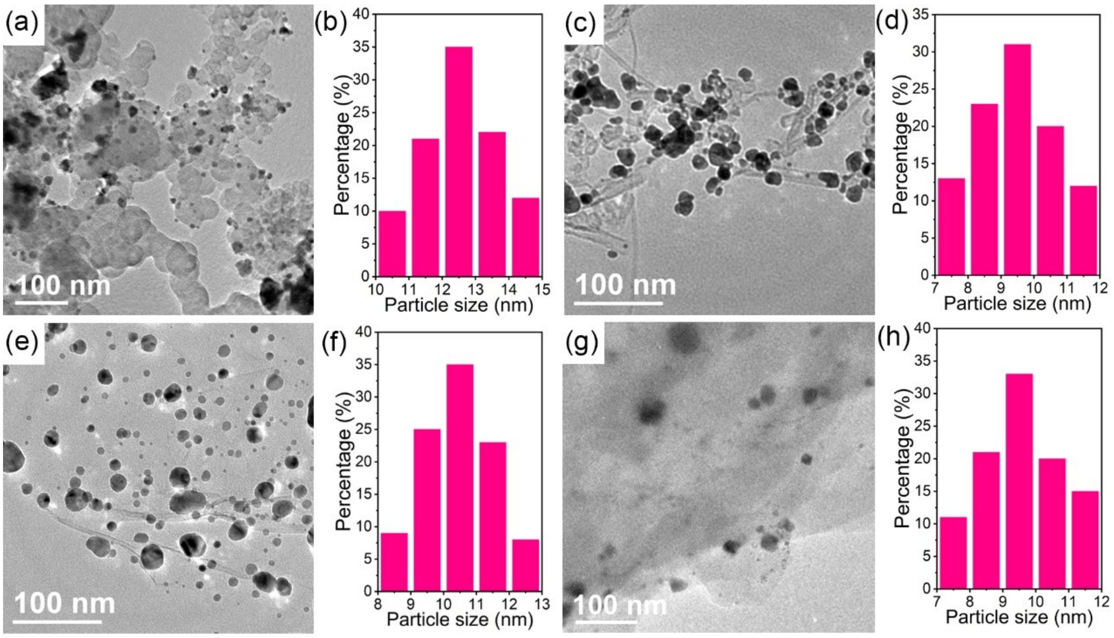

Fig. S6 TEM analysis of the reference hybrid catalysts. Typical TEM images and Pd particle size distribution of (a, b) Pd NPs/C, (c, d) Pd NPs/CNT, (e, f) Pd NPs/RGO, and (g, h) Pd NPs/MXene.

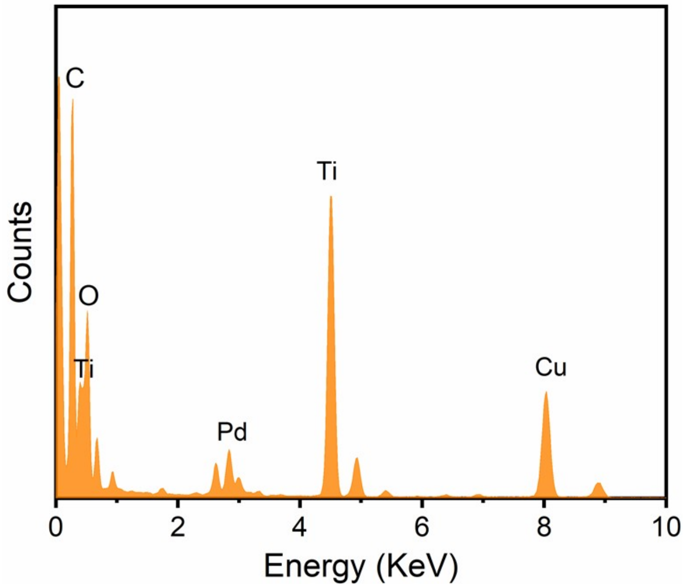

Fig. S7 EDX spectrum of the Pd NSs/MXene catalyst verifies the presence of C, Ti, O, and Pd components in the hybrid. The sample was held on a Cu grid, thus Cu peaks were also detected.

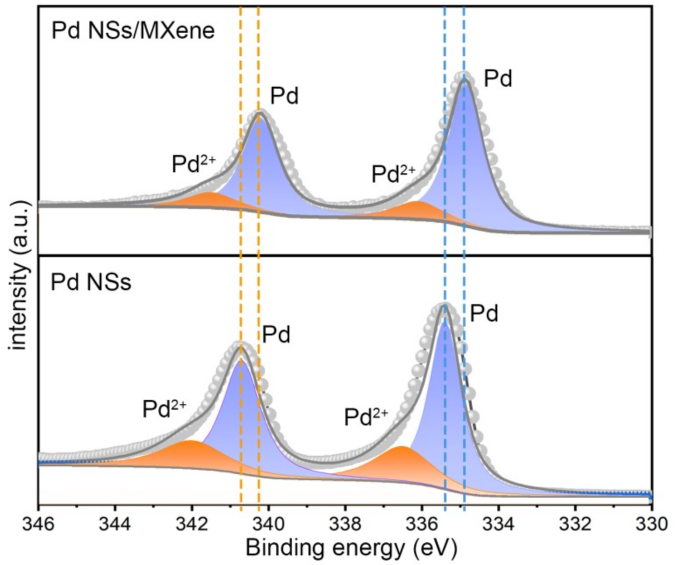

Fig. S8 Comparison of the binding energies for Pd 3d spectra of Pd NSs/MXene with bare Pd NSs.

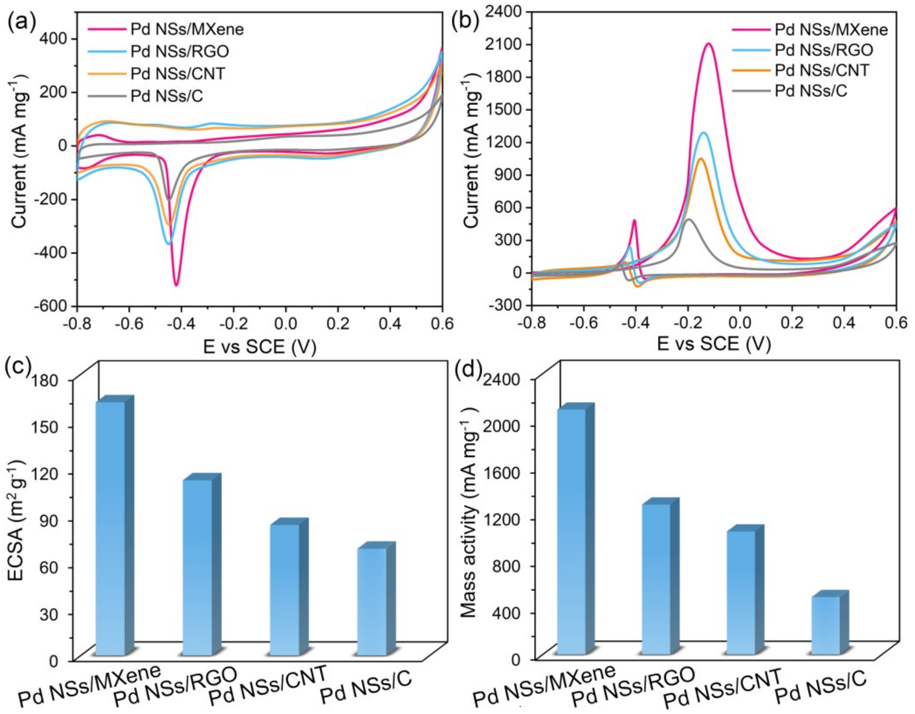

Fig. S9 Typical CV curves of the Pd NSs/MXene, Pd NSs/RGO, Pd NSs/CNT, and Pd NSs/C electrodes in (a) 0.5 M NaOH solution and (b) 0.5 M NaOH with 1 M CH_3OH solution at 50 mV s^{-1}. (c) Specific ECSA values and (d) mass activities for various electrodes.

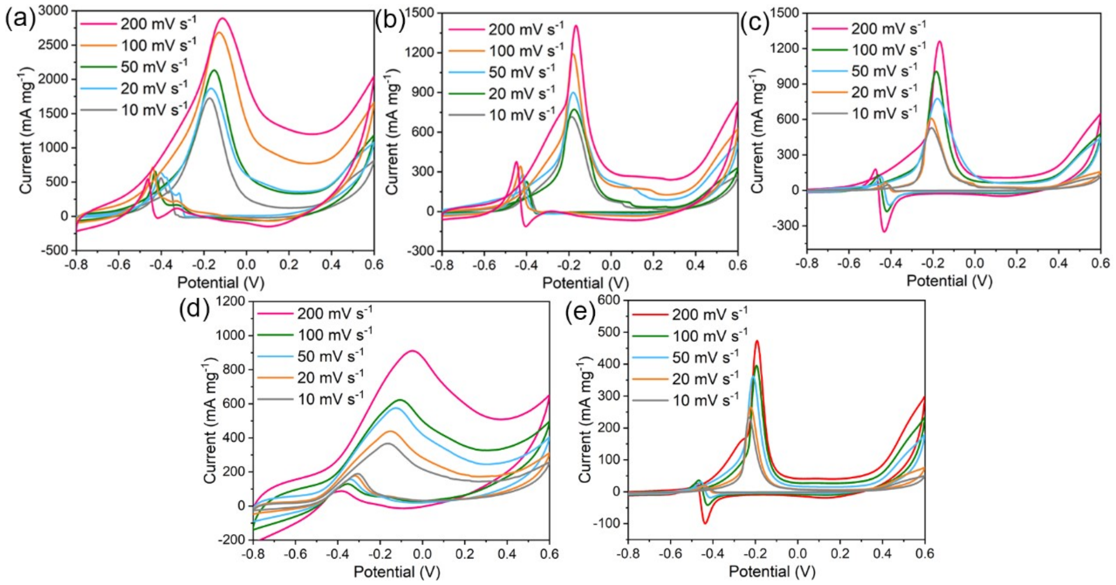

Fig. S10 CV curves of (a) Pd NSs/MXene, (b) Pd NPs/MXene, (c) Pd NPs/RGO, (d) Pd NPs/CNT, and (e) Pd NPs/C in 0.5 M NaOH with 1 M CH_3OH solution with scan rates of 10, 20, 50, 100 and 200 mV s^{-1}.

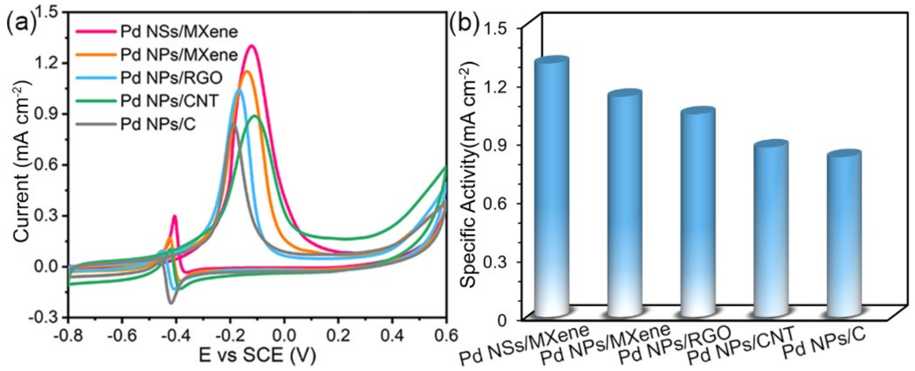

Fig. S11 (a) ECSA-normalized CV curves of the Pd NSs/MXene and other reference catalysts in 0.5 M NaOH with 1 M CH_3OH solution at 50 mV s^{-1}. (b) ECSA-normalized specific activities for different catalysts.

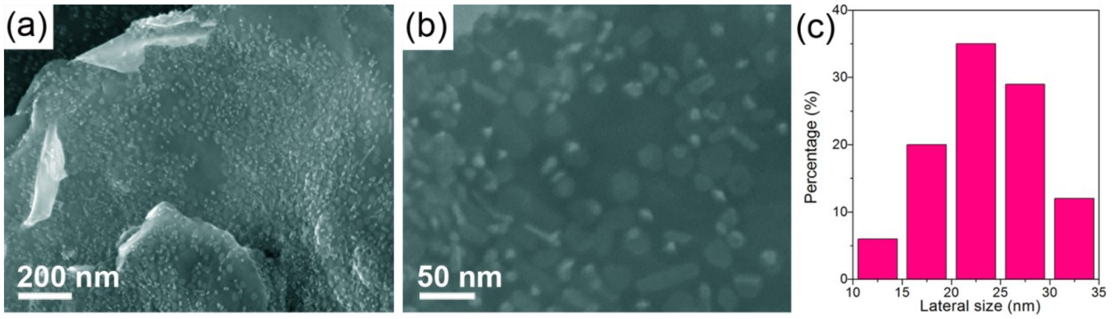

Fig. S12 (a-b) FE-SEM images and the (c) corresponding Pd lateral size distribution of the Pd NSs/MXene catalyst after the long-term durability test.

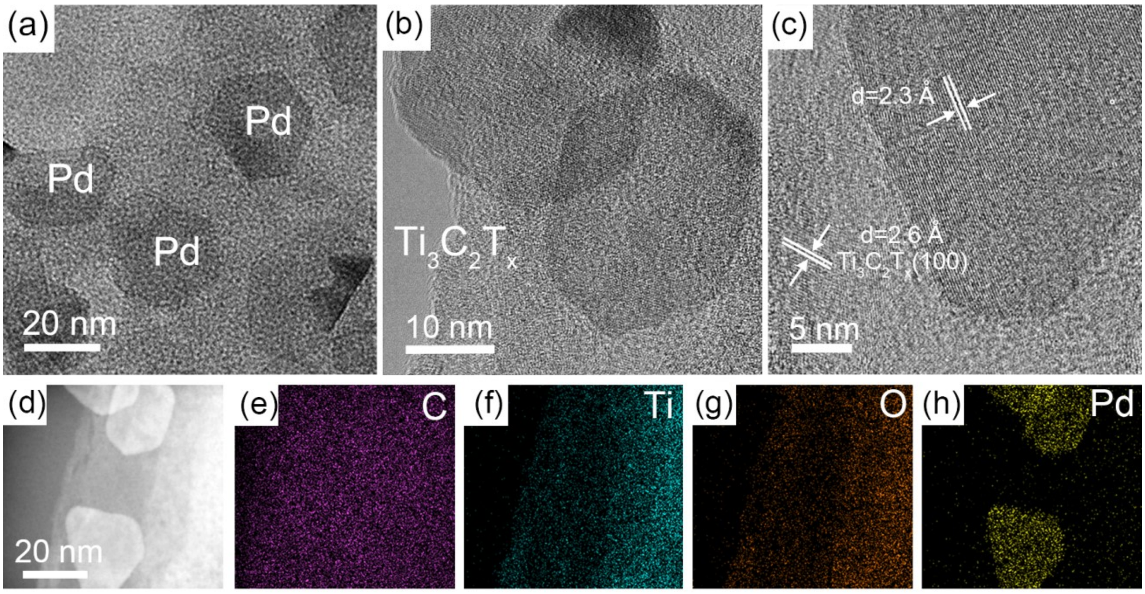

Fig. S13 (a, b) TEM, (c) HR-TEM, (d) HAADF-STEM images and elemental analysis of (e) C, (f) Ti, (g) O, and (h) Pd elements of the Pd NSs/MXene catalyst after the long-term durability test.

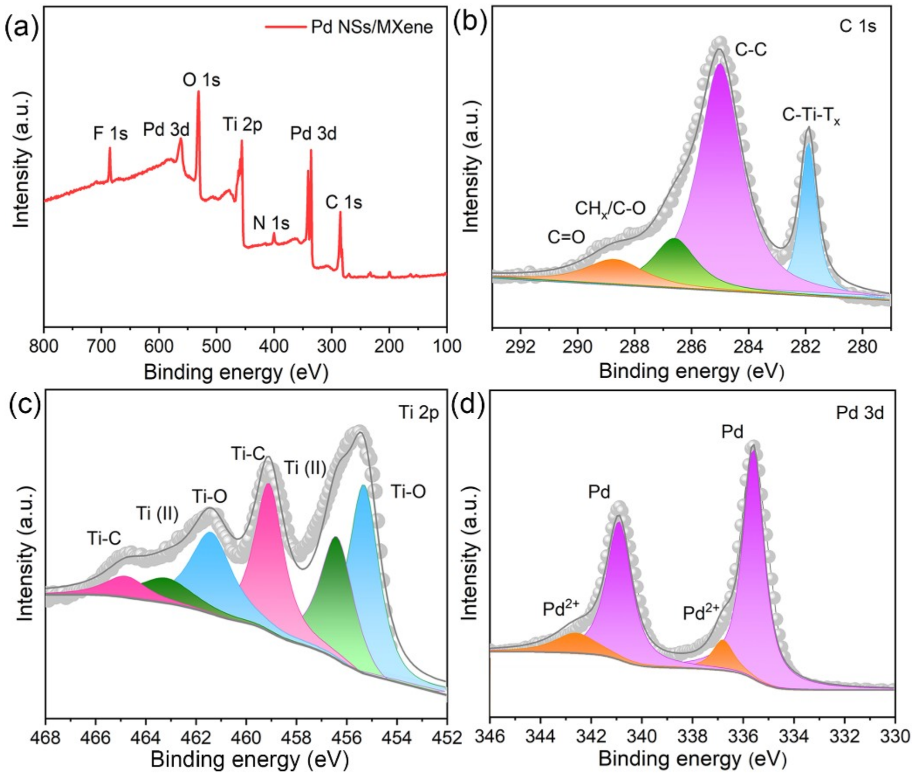

Fig. S14 (a) XPS survey, (b) C 1s, (c) Ti 2p, and (d) Pd 3d core-level spectra of the Pd NSs/MXene catalyst after the long-term durability test.

Table S1. Summary of CV results for different catalysts.

Catalyst	ECSA (m^2 g^{-1})	Mass activity (mA mg^{-1})	Specific activity (mA cm^{-2})	Scan rate (mV s^{-1})
Pd NSs/MXene	162.0	2091.4	1.30	50
Pd NSs/RGO	112.1	1281.5	1.14	50
Pd NSs/CNT	83.5	1049.2	1.25	50
Pd NSs/C	68.3	493.5	0.72	50
Pd NPs/MXene	82.2	934.1	1.13	50
Pd NPs/RGO	75.0	780.9	1.04	50
Pd NPs/CNT	64.2	569.9	0.87	50
Pd NPs/C	41.2	345.5	0.82	50

Table S2. Comparison of methanol oxidation properties for the Pd NSs/MXene catalyst with various recent state-of-the-art Pd-based catalysts.

Catalyst	ECSA ($m^2\ g^{-1}$)	Mass activity ($mA\ mg^{-1}$)	Scan rate ($mV\ s^{-1}$)	Electrolyte	Ref.
Pd NSs/MXene	162.0	2091.4	50	0.5 M NaOH + 1.0 M CH_3OH	This work
PdAg/CNT	27.0	731.0	50	1 M KOH + 0.5 M CH_3OH	49
Pd/APZ-functionalized CNTs	42.3	579.2	50	0.5 M NaOH + 1.0 M CH_3OH	50
Pd/N-doped graphitic carbon nanosheets	136.7	816.5	50	0.5 M NaOH + 1.0 M CH_3OH	51
Pd/N,S-codoped graphene	103.6	399.3	50	0.5 M NaOH + 1.0 M CH_3OH	52
Pd/MnO_2-modified graphene	82.6	838.0	50	0.5 M NaOH + 1.0 M CH_3OH	53
Pd/$CoMoO_4$-modified graphene	50.5	1109.3	50	1 M KOH + 1 M CH_3OH	54
Pd/3D B,N-codoped graphene	82.1	707.5	50	0.5 M NaOH + 1.0 M CH_3OH	33
Pd/3D N-doped graphene-CNT	88.8	1396.0	50	0.5 M NaOH + 1.0 M CH_3OH	31
Pd@RhPd alloy nanodendrites	15.5	437.5	50	1 M KOH + 0.5 M CH_3OH	55
PdCu nanodendrites	24.8	135	50	0.1 M KOH + 0.5 M CH_3OH	56
PdAu nanowires	59.1	N.A.	50	0.5 M NaOH + 1.0 M CH_3OH	57
PdCo nanowires	33.6	1205	50	1 M KOH + 0.5 M CH_3OH	58

Table S3. The charge-transfer resistances of the Pd NSs/MXene, Pd NPs/MXene, Pd NPs/RGO, Pd NPs/CNT, and Pd NPs/C electrodes.

Catalyst	R_{ct} value (Ω)	Error (%)
Pd NSs/MXene	7.9	4.2
Pd NPs/MXene	17.9	5.1
Pd NPs/RGO	21.4	4.8
Pd NPs/CNT	22.1	5.8
Pd NPs/C	6875.1	4.5
